# Some aspects of the acute phase immune response to a lipopolysaccharide (LPS) challenge are mitigated by supplementation with a *Saccharomyces cerevisiae* fermentation product in weaned beef calves^1^,^2^

**DOI:** 10.1093/tas/txaa156

**Published:** 2020-08-24

**Authors:** Nicole C Burdick Sanchez, Jeff A Carroll, P Rand Broadway, Tom S Edrington, Ilkyu Yoon, Craig R Belknap

**Affiliations:** 1 Livestock Issues Research Unit, ARS-USDA, Lubbock, TX; 2 Diamond V, Cedar Rapids, IA

**Keywords:** acute phase response, cattle, cytokines, lipopolysaccharide, *Saccharomyces cerevisiae* fermentation product

## Abstract

This study was conducted to determine if feeding a *Saccharomyces cerevisiae* fermentation product (SCFP) to calves would alter the acute phase response to a lipopolysaccharide (LPS) challenge. Crossbred steer calves [*n* = 32; 274 ± 1.9 kg body weight (BW)] were randomly allotted to two treatment diets for 21 d: 1) control, fed RAMP (Cargill, Dalhart, TX) and 2) SCFP, fed the control ration supplemented with NaturSafe at 12 g/hd/d mixed into the TMR (NaturSafe, Diamond V, Cedar Rapids, IA). On day 22, steers were fitted with indwelling jugular catheters and rectal temperature monitoring devices and placed in individual bleeding stalls. On day 23, steers were challenged i.v. with 0.25 µg/kg BW LPS. Blood samples were collected at 0.5-h (serum) or 2-h (complete blood counts) intervals from −2 to 8 h and again at 24 h relative to the LPS challenge at 0 h. Sickness behavior scores (SBS) were recorded after the collection of each blood sample. Rectal temperatures were greater in SCFP steers from 6 to 11 h, at 13 h, from 15 to 20 h, and from 22 to 24 h following the LPS challenge compared to Control steers (treatment × time: *P* = 0.01). Additionally, SCFP-supplemented steers had reduced (*P* < 0.01) SBS compared to Control steers. Platelet concentrations remained greater in SCFP-supplemented steers compared to Control steers throughout the study (*P* = 0.05), while there was a tendency (*P* = 0.09) for SCFP steers to have greater white blood cells and eosinophils concentrations than Control steers. There was a treatment × time interaction for serum cortisol and glucose (*P* < 0.01). Specifically, cortisol was greater at 0.5 and 2 h postchallenge but was reduced at 3 h for SCFP steers compared to Control steers. Glucose was greater in SCFP steers at −0.5, 2, and 7.5 h compared to Control steers. Serum amyloid A was reduced in SCFP steers at 0.5 h, yet greater at 1 and 7.5 h postchallenge compared to Control steers (treatment × time: *P* < 0.01). Fibrinogen concentrations were greater (*P* < 0.01) in SCFP compared to Control steers. There was a treatment × time interaction (*P* < 0.01) for tumor necrosis factor-α (TNF-α) such that concentrations were reduced in SCFP steers from 1 to 2 h postchallenge compared to Control steers. Overall, these data suggest that supplementing calves with SCFP may have primed the innate immune response prior to the challenge, particularly platelets, which resulted in an attenuated sickness behavior and TNF-α response to LPS.

## INTRODUCTION

There continues to be increased pressure on livestock producers to reduce and/or remove antimicrobials from animal diets from both consumers and federal legislation (i.e., veterinary feed directive; Clark et al., 2012). Additionally, there is a demand from advocacy groups and consumers to produce cattle using more “natural” means, which includes halting the use of antimicrobials, growth implants, and other synthetic supplements in support of using nonsynthetic supplements (Stackhouse et al., 2012; Ovinge et al., 2018). Therefore, there is an increasing number of cattle producers turning to natural or organic production systems (Karreman, 2009). Thus, the need exists to provide producers with viable alternatives to traditional management practices that allow producers to maintain cattle performance and health while helping them align with consumer demand and federal regulations (McEwen and Fedorka-Cray, 2002). Currently, many different feed supplements are being produced and marketed that may be able to fill this need (Kegley and Spears, 1995; Young et al., 2016; Ovinge et al., 2018).

There are many feed supplements that may have the ability to modulate the immune responsiveness of cattle and other livestock species. Various yeast and yeast-based products have been demonstrated to reduce the acute phase response while either not altering or improving performance (Burdick Sanchez et al., 2013; Finck et al., 2014; Broadway et al., 2015; Ovinge et al., 2018). NatureSafe (Diamond V, Cedar Rapids, IA) is a *Saccharomyces cerevisiae* fermentation product (SCFP) that was developed to promote immunity in cattle. Previous studies using SCFP have reported improvements in cattle performance, nutrient digestion, and liver abscesses (Scott et al., 2017; Deters and Hansen, 2019; Shen et al., 2019). However, studies on the effect of SCFP on immunity are limited (Shen et al., 2019). Based on the aforementioned studies, it was hypothesized that supplementation with SCFP would alter the acute inflammatory response to an immune challenge. Therefore, this study was designed to determine if supplementation of receiving cattle with SCFP would alter the acute phase response to a provocative endotoxin (lipopolysaccharide, LPS) challenge.

## MATERIALS AND METHODS

All experimental procedures were in compliance with the *Guide for the Care and Use of Agricultural Animals in Research and Teaching* and approved by the Institutional Animal Care and Use Committee at the Livestock Issues Research Unit (Protocol # 18010F).

### Experimental Design

Crossbred steer calves [(*n* = 32; 274 ± 1.9 kg body weight (BW)] were backgrounded for 45 d prior to transport to a commercial feedlot. Upon feedlot arrival, steers were allotted randomly to two treatment diets fed for 21 d: 1) Control, fed RAMP (Cargill, Dalhart, TX) and 2) SCFP, fed the control diet supplemented with NaturSafe at 12 g/hd/d mixed into the RAMP (NaturSafe, Diamond V, Cedar Rapids, IA). Cattle were randomly allocated to two pens (one pen per treatment) using a computer-generated randomization schedule based on the order of processing. Upon arrival, steers were rested overnight prior to processing, which included: vaccination for respiratory disease pathogens (Bovi-Shield Gold 5, Zoetis, Parsippany-Troy Hills, NJ and Presponse, Boehringer Ingelheim Animal Health USA, Duluth, GA), treatment for internal and external parasites (Ivomec Pour-on, Merial, Duluth, GA and Safe-Guard Drench, Merck Animal Health, Summit, NJ), and placement of an ear tag. Steers were fed three times per day, and treatments were mixed into the total mixed ration (Ramp) fresh at each feeding. The same truck was used for mixing and delivering both rations, with the Control ration fed first at each feeding, then the SCFP ration. Other non-SCFP, nontrial cattle were fed after the SCFP ration was delivered to ensure that residual SCFP was flushed from the truck before the next feeding. After 21 d of supplementation, steers were transported approximately 190 km (1.5 h) to the USDA ARS Livestock Issues Research Unit’s Bovine Immunology Research and Development Complex near New Deal, TX. Upon arrival, steers were placed by treatment in covered outdoor pens (7.6 × 18.3 m; two pens per treatment) with access to respective treatment diets provided in feeding troughs within the pens and water ad libitum overnight. The following day steers were processed through an indoor working facility where the steers were weighed and each fitted with an indwelling jugular catheter for the collection of serial blood samples and a rectal temperature monitoring device (Reuter et al., 2010) that measured rectal temperature at 5-min intervals for the duration of the study. The steers were then moved into individual steel bleeding stalls (length, 2.28 m; width, 0.76 m; and height, 1.67 m) in an enclosed, environmentally controlled barn for the remainder of the study. While in the bleeding stalls, steers had access to an individual waterer within the stall, feed bucket outside the front of the stall, and room for normal maintenance behaviors (standing, lying, eating, etc.). Within treatment, steers were randomly placed in the barn. Following processing, all steers received the remainder of the daily allotment of feed in individual feed buckets placed directly in front of each stall. Stainless steel dividers between stalls prevented steers from consuming feed from adjacent pens. Steers were fed the same diets at the LIRU facility as were fed at the feedlot prior to transportation.

On the following day at 0900 hours (0 h), steers were challenged intravenously with 0.25 µg/kg BW LPS (from *Escherichia coli* O111:B4, Sigma Aldrich, St. Louis, MO). Sickness behavior scores (SBS; described below) were recorded and whole blood samples were collected via the jugular cannula at 0.5-h intervals from −2 to 8 h and again at 24 h relative to the LPS challenge at 0 h for the measurement of serum cortisol, tumor necrosis factor-α (TNF-α), interleukin-6 (IL-6), interferon-γ (IFN-γ), glucose, haptoglobin, serum amyloid A (SAA), and fibrinogen concentrations. Whole blood samples collected for serum isolation were collected in 9-mL collection tubes containing no additive (Starstedt Inc., Newton, NC) and were allowed to clot at room temperature for 0.5 h and were then centrifuged at 1500 × *g* for 20 min at 4 °C. Isolated serum was stored in triplicate aliquots at −80 °C until analyzed. An additional blood sample was collected into 4-mL vacutainers containing EDTA (Fisher Scientific, Pittsburgh, PA) at 2-h intervals from −2 to 8 h and again at 24 h for the measurement of complete blood counts using a ProCyte DX Hematology Analyzer (IDEXX Laboratories, Westbrook, ME) using a bovine-specific algorithm. Respiration rates (RR) were recorded at 2-h intervals from 0 to 8 h and again at 24 h relative to the LPS challenge. For RR, two observers independently measured flank movements for 15 s. The average of the 2 observations was then multiplied by 4 for breaths per minute (BPM).

### Sickness Behavior Scores

A trained observer who was blinded to treatment assessed and recorded each steer’s SBS by visual observation following the collection of each blood sample. Steers were scored on a scale of 1 to 4 (Carroll et al., 2017). Specifically, steers scored as 1 maintained normal maintenance behavior; steers scored as 2 were calm but with head distended and increased respiration; steers scored as 3 displayed clinical signs of sickness, increased respiration and drool, while steers scored as 4 were observed lying on the side with labored breathing and frothing at the mouth. Medical intervention would occur on any steer with an SBS of 4; however, no steer received a score greater than 3 at any time point during the study. Additionally, all steers were displaying normal maintenance behaviors prior to the challenge (i.e., scored a 1); thus, data are presented for scores recorded from 0 to 8 h and again at 24 h.

### Serum Analysis

All serum analyses were performed in duplicate. Cortisol concentrations were determined using a commercially available enzyme immunoassay kit according to the manufacturer’s directions (Arbor Assays, Ann Arbor, MI) by comparison of unknowns to standard curves generated with known concentrations of cortisol. The minimum detectable concentration was 45.4 pg/mL and the intra-assay and interassay coefficients of variation were less than 3.8% and 12.6%, respectively.

Serum cytokine concentrations (TNF-α, IFN-γ, and IL-6) were determined by a custom bovine 2-plex (IL-6 and IFN-γ) and 1-plex (TNF-α) sandwich-based chemiluminescence ELISA kits (Searchlight-Aushon BioSystems, Inc., Billerica, MA). The minimum detectable concentrations were 0.5, 0.1, and 3.3 pg/mL for TNF-α, IFN-γ, and IL-6, respectively. All intra-assay coefficients of variation were less than 16.5% and all interassay coefficients of variation were less than 19.3% for all assays.

Glucose concentrations were determined by a modification of the enzymatic Autokit Glucose (Wako Diagnostics, Richmond, VA) to fit a 96-well format as previously described (Burdick Sanchez et al., 2019). Briefly, 300 µL of the prepared working solution was added to 2 µL of serum or prepared standards in a 96-well plate. Plates were incubated at 37 °C for 5 min and absorption was recorded at 505 nm. The plate reader used for this assay (BioTek Powerwave HT; BioTek Instruments, Winooski, VT) has an incubating and timing feature and, therefore, ensured that the sample absorbances were read immediately following the 5-min incubation. Concentrations of glucose were determined by comparing unknown samples to a standard curve of known glucose concentrations. The minimum detectable concentration was 3.8 mg/dL and the intra-assay and interassay coefficients of variation were less than 13.9% and 12.5%, respectively.

Serum haptoglobin concentrations were determined using a commercially available ELISA kit according to the manufacturer’s directions (Immunology Consultants Laboratory, Inc. Portland, OR). Concentrations of haptoglobin were determined by comparing unknown samples to a standard curve of known haptoglobin concentrations. The minimum detectable concentration was 15.6 ng/mL and the intra-assay and interassay coefficients of variation were less than 8.7% and 15.2%, respectively.

Serum Amyloid A concentrations were determined using a commercially available ELISA kit according to the manufacturer’s directions (Thermo Fisher Scientific, Waltham, MA). Concentrations of SAA were determined by comparing unknown samples to a standard curve of known SAA concentrations. The minimum detectable concentration was 1.5 µg/mL and the intra-assay and interassay coefficients of variation were less than 8.9% and 3.5%, respectively.

Serum fibrinogen concentrations were determined using a commercially available ELISA kit according to the manufacturer’s directions (MyBioSource, San Diego, CA). Concentrations of fibrinogen were determined by comparing unknown samples to a standard curve of known fibrinogen concentrations. The minimum detectable concentration was 0.25 mg/mL and the intra-assay and interassay coefficients of variation were 4.1% and 20.3%, respectively.

### Statistical Analysis

Prior to analysis, rectal temperature data were averaged into 1-h intervals. Data were analyzed as repeated measure over time with the use of the MIXED procedure of SAS (SAS Inst. Inc., Cary, NC; v. 9.4). Treatment, time, and the treatment × time interaction were included as fixed effects with steer within treatment included as the experimental unit. Autoregressive 1 covariance structure was used based on having the lowest AIC fit statistic value. When main effects were significant, means were separated using the PDIFF option in SAS, with *P* ≤ 0.05 considered significant and trends toward significance considered at 0.05 < *P* ≤ 0.10. All data are presented as the least squares means (LSM) ± SEM.

## RESULTS

### Physiological Measurements

There was no effect of treatment on steer BW (*P* = 0.41) measured on the day immediately prior to and following the LPS challenge. Steers weighed 310 versus 317 ± 5 kg for Control and SCFP treatments, respectively, on the day of cannulation (1 d prior to the LPS challenge). There was a tendency (*P* = 0.06) for steers to lose weight during the study, as expected, with steers weighing 308 versus 311 ± 5 kg for Control and SCFP steers, respectively, on the day following the LPS challenge. There was no treatment × time interaction for steer BW (*P* = 0.33). Additionally, there was no effect (*P* = 0.48) of treatment on feed disappearance for the approximately 48 h the steers were housed in bleeding stalls in the barn.

**Figure 1.**
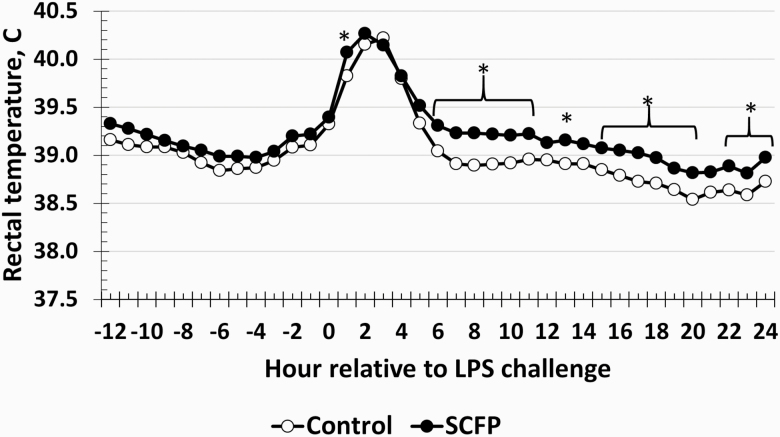
Influence of supplementing steers with an SCFP (12 g/hd/d; *n* = 16) or not (Control; *n* = 16) for 21 d on the rectal temperature response to LPS (0.25 µg/kg BW) challenge. Rectal temperature was measured via indwelling rectal temperature monitoring devices from −12 to 24 h at 5-min intervals and averaged into 1-h intervals prior to analysis. There was a treatment × time interaction (*P* = 0.01) where rectal temperature was greater in SCFP-supplemented steers at 1 h, from 6 to 11 h, at 13 h, from 15 to 20 h, and from 22 to 24 h (*P* ≤ 0.05). *Treatments differ *P* ≤ 0.05. SEM ± 0.08 °C.

**Figure 2.**
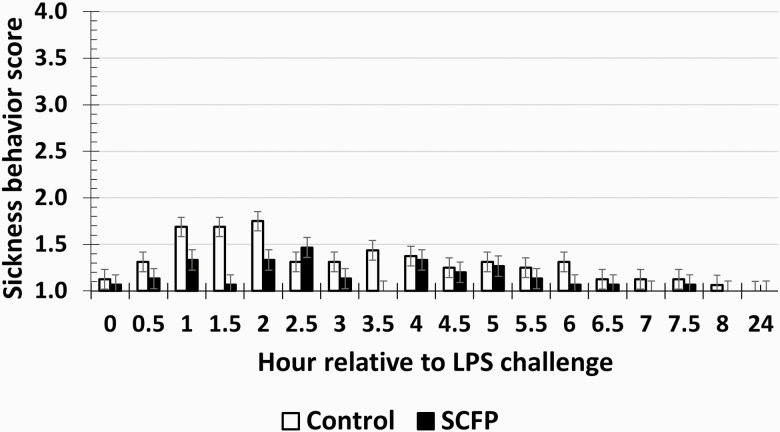
Influence of supplementing steers with an SCFP (12 g/hd/d; *n* = 16) or not (Control; *n* = 16) for 21 d on the sickness behavior score response to LPS (0.25 µg/kg BW) challenge. Sickness behavior scores were recorded on a scale of 1 (normal maintenance behaviors) to 4 (laying on side with labored breathing) every 0.5 h from −2 to 8 h relative to the administration of LPS at 0 h. There was a tendency (*P* = 0.07) for a treatment × time interaction such that SCFP steers had reduced SBS from 1 to 2 h and at 3.5 h postchallenge. Sickness behavior scores were greater (*P* < 0.01) in Control steers than SCFP-supplemented steers.

There was a treatment × time interaction (*P* = 0.01) for rectal temperature such that rectal temperature was greater in SCFP-supplemented steers compared to Control steers at 1 h, from 6 to 11 h, at 13 h, from 15 to 20 h, and from 22 to 24 h (*P* ≤ 0.05; Fig. 1). There was a tendency (*P* = 0.07) for a treatment × time interaction for SBS such that SCFP steers had reduced SBS from 1 to 2 h and at 3.5 h postchallenge. However, overall SBS in both treatments were relatively low throughout the study (Fig. 2). There was no treatment × time interaction (*P* = 0.70) or an effect of treatment (*P* = 0.55) on RR measured every 2 h from 0 to 8 h and again at 24 h relative to the LPS challenge at 0 h. The RR for Control averaged 57.5 ± 1.4 BPM, while SCFP-supplemented steers averaged 58.7 ± 1.4 BPM. Additionally, there were fluctuations in RR over time (*P* < 0.01).

### Complete Blood Counts

**Figure 3.**
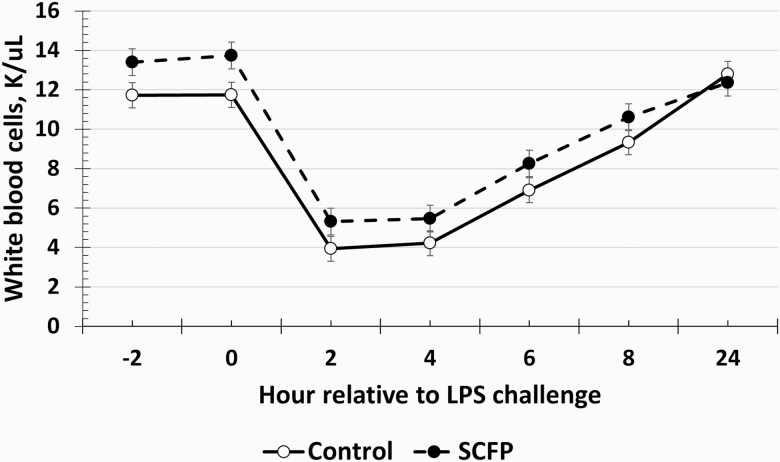
Influence of supplementing steers with an SCFP (12 g/hd/d; *n* = 16) or not (Control; *n* = 16) for 21 d on the white blood cell response to LPS (0.25 µg/kg BW) challenge. Complete blood counts were measured on whole blood samples collected every 2 h from −2 to 8 h and again at 24 h relative to LPS challenge at 0 h. There was a tendency (*P* = 0.09) for greater white blood cell concentrations in SCFP compared to control steers.

There was no treatment × time interaction for any of the hematology parameters measured (*P* ≥ 0.10). There were no treatment effects for red blood cells, hemoglobin, or hematocrit (*P* ≥ 0.48; Table 1; Supplementary Fig. S1), although these parameters changed over time (*P* < 0.01). Specifically, the values of these three parameters increased within 2 h following the LPS challenge but returned to baseline values within 6–8 h of the challenge. Platelet concentrations remained elevated in SCFP-supplemented steers compared to Control steers throughout the study (treatment *P* = 0.05; Supplementary Fig. S1). There was also a time effect (*P* < 0.01) such that platelet concentrations decreased following the LPS challenge and did not return to baseline values by 24 h postchallenge. There was no treatment × time interaction for platelet concentrations (*P* = 0.89). There was a tendency (*P* = 0.09) for a treatment effect on total white blood cell concentrations, where SCFP-supplemented steers tended to have greater white blood cell concentrations compared to Control steers (Fig. 3). White blood cell concentrations decreased within 2 h following the LPS challenge, which recovered within 24 h (time: *P* < 0.01). Additionally, white blood cell concentrations were greater in SCFP-supplemented steers prior to the challenge (−2 to 0 h; treatment: *P* = 0.05). Due to the difference in baseline values, the change in white blood cell concentrations, relative to average baseline values, was analyzed, which found no effect of treatment (*P* = 0.36). A similar time effect (*P* < 0.01) was observed for neutrophil and lymphocyte concentrations, although there were no treatment effects observed for these variables (*P* ≥ 0.21). As expected, based on neutrophil and lymphocyte concentrations, there was a time effect for the neutrophil:lymphocyte ratio (*P* < 0.01), but there was no treatment effect (*P* = 0.17). Monocyte concentrations also followed a similar temporal pattern as the other immune cell populations, with a decrease within 2 h postchallenge and recovery within 24 h (time: *P* < 0.01); however, there was no treatment effect (*P* = 0.16). Eosinophils tended (*P* = 0.09) to be greater in SCFP-supplemented steers. Additionally, eosinophil concentrations increased within 2 h following the LPS challenge and remained elevated through 24 h postchallenge (time: *P* < 0.01). Data from white blood cell differential counts are presented in Supplementary Fig. S2.

**Table 1.** Influence of supplementing steers with SCFP (12 g/hd/d; *n* = 12) or not (Control; *n* = 12) for 21 d on the complete blood count response to LPS (0.25 µg/kg BW) challenge^*a*,*b*^

				*P*-value		
Variable	Control	SCFP	SEM	Treatment	Time	Interaction^*c*^
Red blood cells, M/µL	8.06	8.09	0.144	0.87	<0.01	0.37
Hemoglobin, g/dL	11.0	10.9	0.180	0.57	<0.01	0.10
Hematocrit, %	33.7	33.0	0.65	0.48	<0.01	0.49
Platelets, K/µL	457	537	27.9	0.05	<0.01	0.89
White blood cells, K/µL	8.67	9.88	0.499	0.09	<0.01	0.26
Neutrophils, K/µL	2.97	3.58	0.342	0.21	<0.01	0.67
Lymphocytes, K/µL	4.35	4.57	0.357	0.35	<0.01	0.20
Neutrophil:lymphocyte	0.679	0.842	0.0814	0.17	<0.01	0.65
Monocytes, K/µL	1.29	1.45	0.077	0.16	<0.01	0.37
Eosinophils, K/µL	0.134	0.208	0.0301	0.09	<0.01	0.72

^*a*^SCFP: NaturSafe Diamond V, Cedar Rapids, IA.

^*b*^Whole blood samples for measurement of complete blood counts were collected every 2 h from −2 to 8 h and again at 24 h relative to the LPS challenge at 0 h.

^*c*^Interaction: treatment × time.

### Serum Cortisol and Glucose

**Figure 4.**
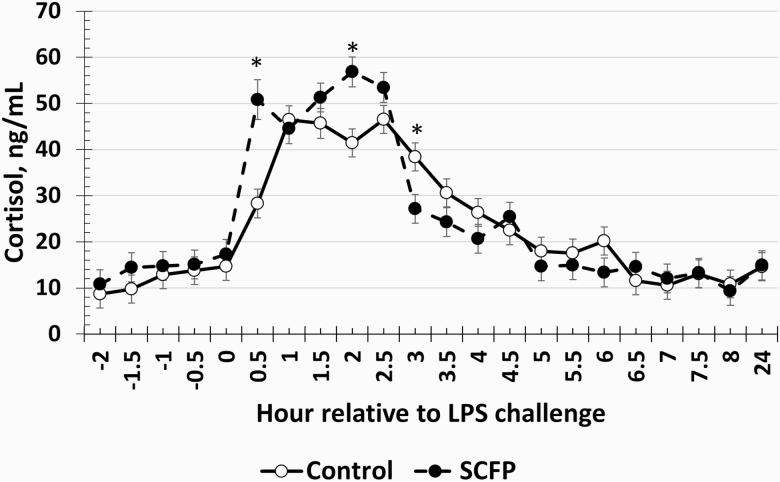
Influence of supplementing steers with an SCFP (12 g/hd/d^-^; *n* = 16) or not (Control; *n* = 16) for 21 d on the cortisol response to LPS (0.25 µg/kg BW) challenge. Serum cortisol concentrations were measured in serum samples collected every 0.5 h from −2 to 8 h and again at 24 h relative to LPS administration at 0 h. There was a treatment × time interaction (*P* < 0.01), where cortisol concentrations were greater in SCFP-supplemented steers compared to Control steers at 0.5 and 2 h postchallenge yet were reduced in SCFP compared to Control steers at 3 h postchallenge. *Treatments differ *P* ≤ 0.01.

**Figure 5.**
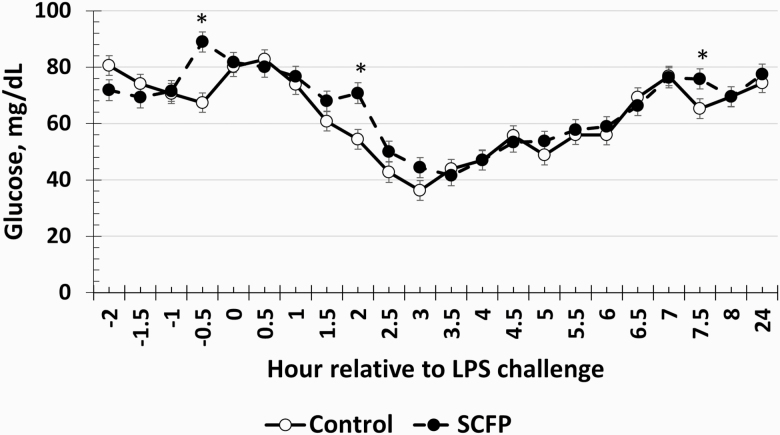
Influence of supplementing steers with an SCFP (12 g/hd/d; *n* = 16) or not (Control; *n* = 16) for 21 d on the glucose response to LPS (0.25 µg/kg BW) challenge. Serum glucose concentrations were measured in blood samples collected every 0.5 h from −2 to 8 h and again at 24 h relative to administration of LPS at 0 h. There was a treatment × time interaction (*P* = 0.01), where SCFP-supplemented steers had greater glucose concentrations at −0.5, 2, and 7.5 h following LPS challenge. *Treatments differ *P* ≤ 0.05.

There was a treatment × time interaction (*P* < 0.01) for serum cortisol concentrations (Fig. 4). Specifically, cortisol concentrations were greater in SCFP-supplemented steers compared to Control steers at 0.5 and 2 h postchallenge yet were reduced in SCFP compared to Control steers at 3 h postchallenge. There was also a treatment × time interaction (*P* = 0.01) for serum glucose concentrations (Fig. 5). SCFP-supplemented steers had greater glucose concentrations at –0.5, 2, and 7.5 h following LPS challenge.

### Serum Cytokines

**Figure 6.**
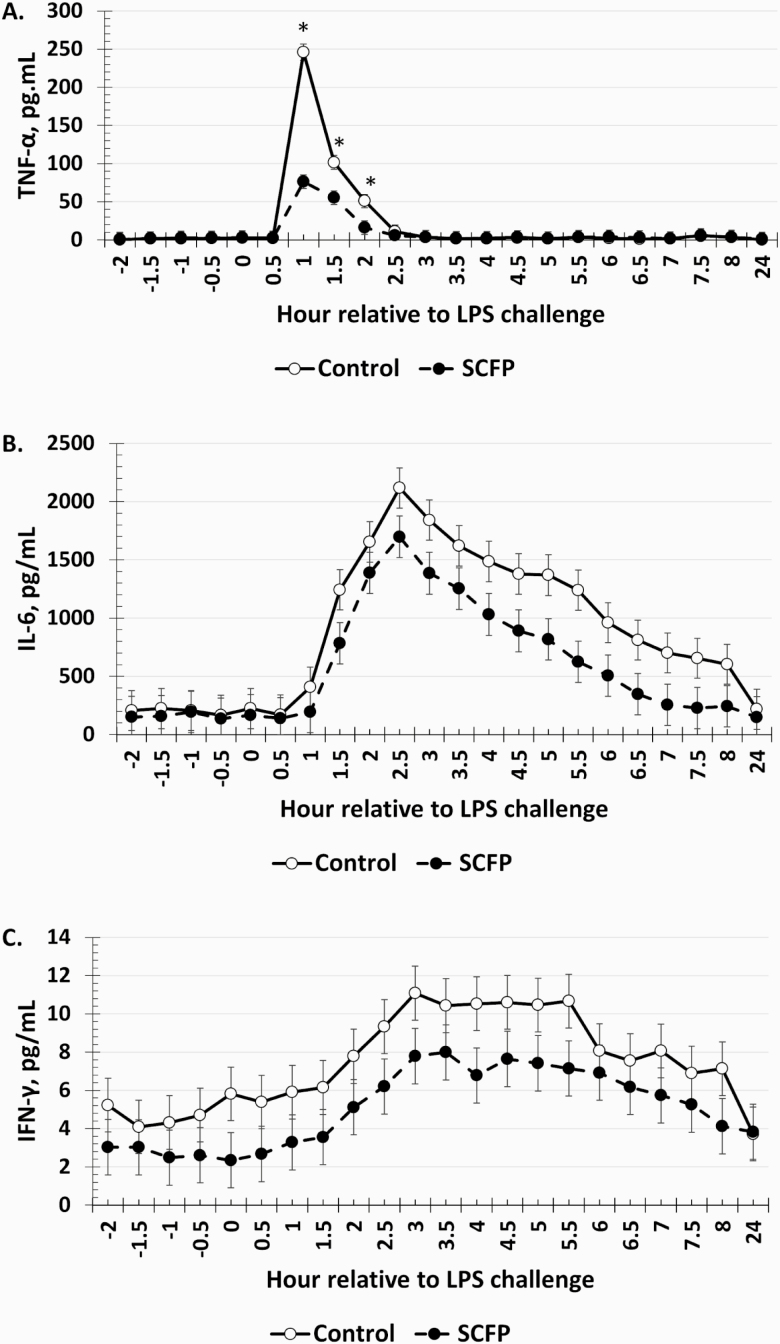
Influence of supplementing steers with an SCFP (12 g/hd/d; *n* = 16) or not (Control; *n* = 16) for 21 d on the (A) TNF-α; (B) IL-6; and (C) IFN-γ responses to LPS (0.25 µg/kg BW) challenge. Serum cytokine concentrations were measured in blood samples collected every 0.5 h from −2 to 8 h and again at 24 h relative to LPS administration at 0 h. There was a treatment × time interaction (*P* < 0.01) for TNF-α concentrations (A), which were reduced in SCFP-supplemented steers compared to Control steers from 1 to 2 h postchallenge. Serum IL-6 concentrations (B) tended (*P* = 0.09) to be reduced in SCFP steers compared to Control steers. A tendency (*P* = 0.07) existed for a treatment × time interaction for serum IFN-γ. *Treatments differ *P* ≤ 0.003.

There was a treatment × time interaction (*P* < 0.01) for serum TNF-α concentrations (Fig. 6A). Specifically, TNF-α concentrations were reduced in SCFP-supplemented steers compared to Control steers from 1 to 2 h postchallenge. There was no treatment × time interaction for IL-6 (*P* = 0.35). Serum IL-6 concentrations tended (*P* = 0.09) to be reduced in SCFP steers compared to Control steers (579 vs. 887 ± 127 pg/mL; Fig. 6B). Concentrations of IL-6 increased within 1.5 h following the LPS challenge and remained elevated until the collection of the 24 h sample (time: *P* < 0.01). There was a tendency (*P* = 0.07) for a treatment × time interaction for serum IFN-γ concentrations (Fig. 6C). Specifically, IFN-γ concentrations tended (*P* ≤ 0.10) to be reduced in SCFP steers at 0, 3, 4, and 5.5 h relative to the LPS challenge.

### Serum Acute-Phase Proteins

**Figure 7.**
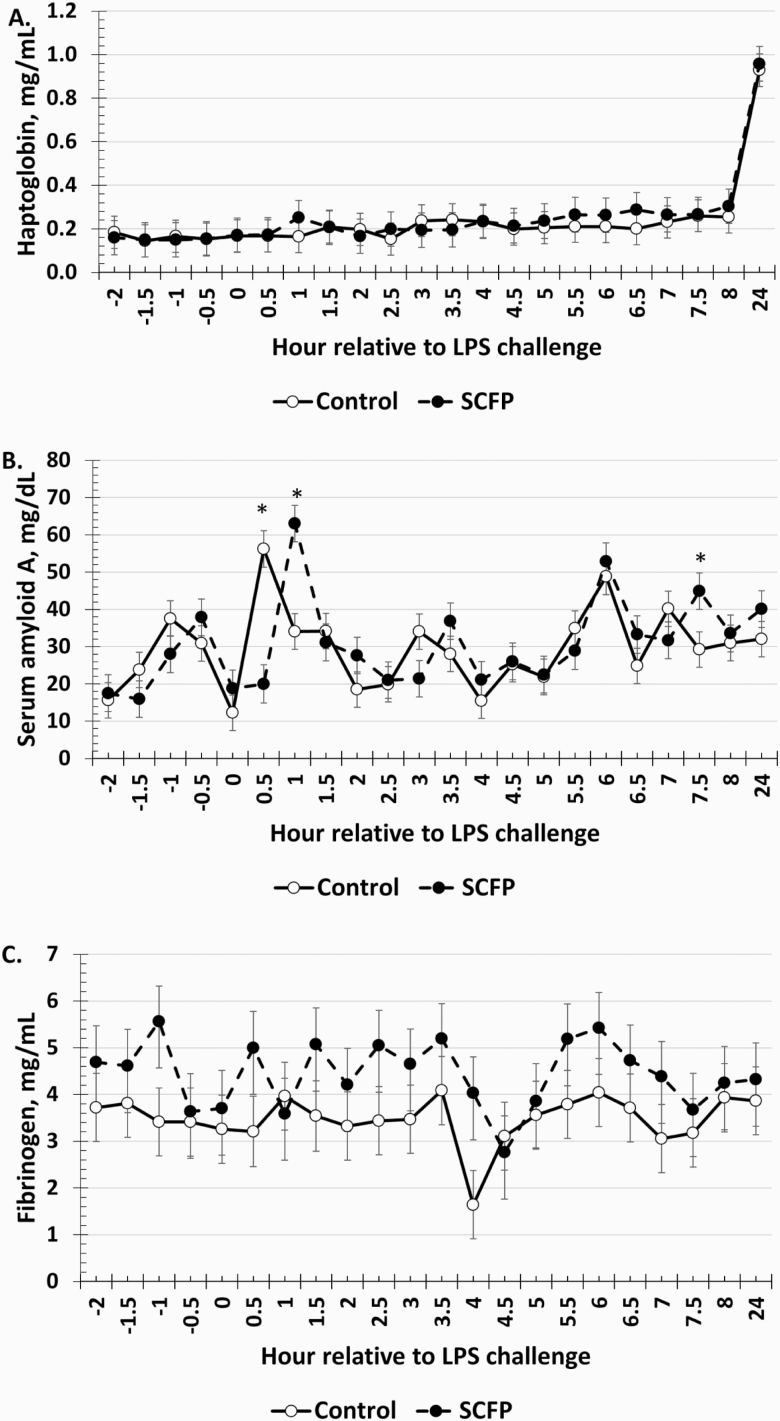
Influence of supplementing steers with an SCFP (12 g/hd/d; *n* = 16) or not (Control; *n* = 16) for 21 d on the (A) haptoglobin; (B) SAA; and (C) fibrinogen responses to LPS (0.25 µg/kg BW) challenge. Serum acute phase proteins were measured in blood samples collected every 0.5 h from −2 to 8 h and again at 24 h relative to LPS administration at 0 h. Serum haptoglobin concentrations increased over time (*P* < 0.01; A). There was a treatment × time interaction (*P* < 0.01) for SAA concentrations (B), where concentrations were greater in Control steers at 0.5 h but were greater in SCFP steers at 1 and 7.5 h postchallenge. Fibrinogen concentrations (C) were greater (*P* < 0.01) in SCFP-supplemented steers than Control steers. *Treatments differ *P* ≤ 0.02.

Serum haptoglobin concentrations were not affected by a treatment × time interaction or by treatment (*P* ≥ 0.81); however, there was an effect of time (*P* < 0.01; Fig. 7A). Specifically, haptoglobin concentrations began a gradual increase at 5.5 h postchallenge, peaking at 24 h postchallenge. There was a treatment × time interaction (*P* < 0.01) for SAA concentrations (Fig. 7B). Specifically, steers supplemented with SCFP had a delayed peak SAA response compared to Control calves, which resulted in reduced concentrations in SCFP steers than Control steers at 0.5 h but an opposite response at 1 h where SCFP calves had greater SAA than Control calves. There was no effect of time or a treatment × time interaction (*P* ≥ 0.60) for serum fibrinogen concentrations. However, fibrinogen concentrations were greater (*P* < 0.01; Fig. 7C) in SCFP steers compared to Control steers.

## DISCUSSION

Lipopolysaccharide is a component of the cell wall of gram-negative bacteria and, thus, is used to mimic an acute bacterial infection. Utilization of an LPS challenge enables researchers to study the effects of treatments on the innate immune response/acute phase response to an acute immune stimulus, which causes moderate morbidity without causing mortality, making an LPS challenge model advantageous compared to a live pathogen challenge or experimental infection. The acute LPS challenge stimulates increases in various inflammatory mediators over a short duration of time, with resolution typically within 24 h, depending on the dose used.

One aspect of the acute phase response to LPS is a rapid increase in body temperature following administration. This is likely a result of increasing concentrations of pyrogenic pro-inflammatory cytokines, such as TNF-α, IL-1, and IL-6, which affect the thermoregulatory centers of the brain in response to an infection (Lohuis et al., 1988; Dinarello, 1996). Interestingly, rectal temperature was greater in SCFP-supplemented steers, while TNF-α and IL-6 concentrations were decreased in this treatment. It is unclear what is driving the increased rectal temperature in supplemented steers. There are varying responses in pigs and cattle supplemented with other SCFP. A study utilizing a similar SCFP in weaned pigs reported greater baseline intraperitoneal temperature in supplemented pigs compared to control pigs prior to an LPS challenge; however, the change in intraperitoneal temperature following the challenge was not affected by supplementation with the similar SCFP (Burdick Sanchez et al., 2018). A study in pigs challenged with *Salmonella* found no effect of SCFP supplementation when rectal temperature was measured weekly (Price et al., 2010), while dairy calves supplemented with an SCFP and challenged with *Salmonella* had decreased rectal temperature compared to control calves when rectal temperature was recorded daily (Brewer et al., 2014). Additionally, lactating dairy cows were also observed to have decreased rectal temperature when supplemented with an SCFP and exposed to heat stress compared to nonsupplemented control cows (Zhu et al., 2016). Therefore, there are differences in the effects of SCFP depending on the animal and challenge model used. One important difference with the current study compared to the aforementioned studies was that rectal temperature was measured frequently using indwelling temperature recording devices, allowing the recording of temperature in the absence of human intervention, which likely allows for a more accurate measure of body temperature.

It is interesting that the greater rectal temperature values observed in SCFP compared to Control steers were not observed until after the peak temperature response to the LPS challenge. Specifically, they were observed between 6 and 24 h following the challenge when values had returned to baseline. While there may only be a couple tenths of a degree difference in the rectal temperatures between treatments, it is known that an increase in body temperature 1 °C can result in a 10–13% increase in metabolizable energy, while more recent data suggests that the energy demand is much greater (Kluger and Rothenburg, 1979; Humphrey and Klasing, 2004; Huntley et al., 2017). Additionally, the lack of adequate energy reserves can result in a hypothermic response rather than a hyperthermic response (Carroll et al., 2001), which may be more important considering the gradual decrease in body temperature exhibited by both treatments postchallenge. Thus, while the differences in body temperature may be small, there may be a significant biological impact on energy availability. It is possible that differences in energy availability are playing a role in the greater rectal temperature in SCFP-supplemented steers, which is discussed further below. Regardless, the difference in body temperature between Control and SCFP-supplemented steers in response to an LPS challenge is an area that requires further study.

The increased rectal temperature observed in the current study, when observed with the other parameters measured (and discussed below), suggests that this is a positive response of the supplement. Perhaps the increased rectal temperature is a means to reduce bacterial growth, as greater body temperatures have been demonstrated to reduce bacterial growth and improve animal survival (Kluger and Rothenburg, 1979; Klasing, 1988; Kluger et al., 1998). However, increased body temperature for an extended period of time may have negative effects on energy stores due to the effect of fever on energy utilization (Loew, 1974; Carroll and Forsberg, 2007; Kvidera et al., 2017). Yet, as discussed below with regards to glucose, it is also possible that SCFP is altering metabolism or has a glucose-sparing effect, which may impact body temperature.

The same cytokines (TNF-α and IL-1) that influence body temperature also stimulate SBS (Dantzer, 2001, 2004). In contrast to rectal temperature, the reduced sickness behaviors observed in SCFP-supplemented steers corresponds with the reduced concentrations of TNF-α and IL-6 observed in these steers. Specifically, the reduced SBS observed from 1 to 2 h postchallenge occurred at the same time that reduced concentrations of TNF-α concentrations were observed. However, overall SBS were low, potentially a reflection of the LPS dose. Doses of LPS administered to cattle have ranged from 2 ng/kg to 2 µg/kg BW in published literature and also utilize different serotypes of LPS, which may have different potencies (Waldron et al., 2003; Jacobsen et al., 2005; Carroll et al., 2009). Our laboratory typically utilizes a dose of LPS between 0.25 and 0.5 µg/kg BW, selected based on the background and breed of the cattle, and has used the same serotype of LPS (O111:B4). In the current study, the 0.25 µg/kg BW dose was chosen in order to elicit an adequate inflammatory response to decipher if treatment differences were present. Utilization of a greater dose, which can cause extensive inflammation, may mask any treatment differences present and, thus, there must be a balance between stimulating enough of an immune response without causing too much inflammation. While there were differences in SBS between Control and SCFP treatments, it is possible that the differences observed were not biologically significant due to the fact that the average SBS remained below a score of 2. Nonetheless, the SBS data support other variables reported herein. Respiration rates were not affected by SCFP supplementation. It is possible that differences existed in respiration rate, but these differences were not detected based on the time frame that RR was recorded.

The post-LPS decline in circulating platelets, white blood cells, and differentials was expected and is a normal response to LPS challenge (Jacobsen et al., 2005; Burdick Sanchez et al., 2018). This decrease in circulating immune cells occurs as the immune cells leave circulation and extravasate into tissues in search of the infection (Middleton et al., 2002). Recovery was observed in most complete blood cell variables within 24 h, typical of the acute nature of an LPS challenge. Of the complete blood count variables measured, greater concentrations were observed for platelets, and there was a tendency for greater white blood cells and eosinophils in SCFP-supplemented steers. While platelets are often associated with blood clotting and plaque formation, platelets also play a role in the inflammatory response, releasing several chemokines involved in chemoattraction and activation of leukocytes, such as neutrophils and monocytes (Klinger, 1997). These roles suggest that the increase in circulating platelets in SCFP-supplemented steers may be a means of priming the immune system such that the immune system is prepared for interaction with a pathogen. The greater platelet concentrations observed in the current study are supported by a trend for an increase in platelets in weaned beef steers supplemented with SCFP (Deters and Hansen, 2019). White blood cell concentrations were greater in SCFP steers compared to Control steers prior to the challenge and tended to be greater throughout the entire challenge period. The fact that no treatment differences were observed in the change in white blood cell concentrations suggests that, while there were differences in baseline concentrations, the overall magnitude of the response to the challenge was similar between SCFP and Control steers. Similar to platelets, it is hypothesized that these greater leukocyte concentrations are due to a priming of the innate immune system by SCFP. The greater number of immune cells circulating in the blood (approximately 17% more platelets and 14% more white blood cells) may result in a quicker detection of, and response to, an invading pathogen (Benschop et al., 1996). The greater number of immune cells can be likened to a greater number of infantry troops. This greater number of infantry troops in the body can stop pathogens faster and with greater force than if there were fewer troops present. This priming effect allowed for a reduced pro-inflammatory response as supported by the reduced SBS and pro-inflammatory cytokine concentrations in the steers supplemented with SCFP, discussed below.

Cortisol plays an important role in the pro-inflammatory response. Normal cortisol concentrations are typically between 10 and 20 ng/mL throughout the day under nonstressful conditions, as indicated by the baseline values observed prior to LPS administration. While cortisol initially helps to stimulate the pro-inflammatory response, continued elevation of cortisol concentrations helps prevent a hyperinflammatory state that could exacerbate recovery and cause further damage to healthy tissue (Chinenov and Rogatsky, 2007). The greater concentrations of cortisol early in the inflammatory response (0.5–2 h postchallenge) support the hypothesis that steers supplemented with SCFP were better prepared for an infection, resulting in a response to the LPS challenge that was greater in magnitude but of less duration compared to Control steers. Additionally, cortisol concentrations appeared to resolve earlier in SCFP-supplemented steers than in Control steers as indicated by the decrease in cortisol in the SCFP group at 3 h postchallenge compared to Control steers, which, as discussed earlier, is important in preventing a hyperinflammatory state. The increased cortisol concentrations in SCFP-supplemented steers coincide with greater glucose concentrations in SCFP-supplemented steers at 2 h postchallenge. Cortisol is a glucocorticoid that plays a leading role in the release of glucose from glycogen stores and in the production of glucose via gluconeogenesis (Long et al., 1940). Additionally, the greater cortisol concentrations may be in part responsible for the reduced cytokine concentrations observed. A study where dairy cows were supplemented with an SCFP prior to and following parturition observed reduced cortisol concentrations in supplemented compared to control cows (Zaworski et al., 2014), which supports the cortisol concentrations observed in the current data.

As was observed in this study, glucose concentrations initially increase in response to an LPS challenge, which is stimulated by cortisol and catecholamines to provide energy for the immune system (Lang et al., 1985). The subsequent decrease in glucose concentrations is a result of glucose utilization by the immune system, which is used faster than can be released by glycogenolysis and gluconeogenesis. The greater glucose concentrations measured in the SCFP-supplemented steers indicates that these steers may not have needed to utilize as much glucose in order to recover from the inflammatory insult as did Control steers. Therefore, steers supplemented with SCFP may have altered energy availability, which has been observed with other yeast-based supplements and requires further study (Burdick Sanchez et al., 2019). Furthermore, it suggests no negative effect of the greater rectal temperature observed in SCFP-supplemented steers on energy availability, as glucose concentrations were greater in SCFP-supplemented steers even while rectal temperature remained greater than Control steers. It is possible that the greater energy availability, perhaps due to reduced inflammation or due to greater heat of rumen fermentation, resulted in the greater rectal temperature observed in SCFP-supplemented steers. Additionally, increased energy utilization by Control steers, as indicated by reduced glucose concentrations, may have reduced the amount of energy available for the production of heat, causing the reduced temperature values observed. Work in pigs has shown that neonatal pigs do not produce a febrile response to LPS due to the lack of energy stores available (Carroll et al., 2001). In support of this, studies by Kvidera et al. (2016) and Kvidera et al. (2017) have demonstrated that lactating cows and beef steers can use over 1 kg of glucose within 12 h during an LPS challenge. Thus, while the greater glucose concentrations observed in SCFP-supplemented steers may appear small, these changes may have significant biological effects on other aspects of the acute phase response.

A study by Shen et al. (2019), utilizing SCFP, reported an increase in glucose concentrations in finishing steers 28 d after the start of supplementation compared to nonsupplemented control steers. It is possible that the greater propionate production in the rumen, stimulated by SCFP supplementation (Zhu et al., 2017), played a role in the greater glucose production observed in the current study. Additionally, Zaworski et al. (2014) observed greater glucose concentrations in dairy cows following parturition when supplemented with an SCFP. Thus, there may also be a glucose-sparing effect (due to a reduced pro-inflammatory response) or overall effect of SCFP on energy availability and utilization (i.e., increased ruminal propionate production) in cattle that requires further study.

Overall, the pro-inflammatory cytokine response was reduced by SCFP supplementation. Peak TNF-α concentrations were reduced over 3-fold in SCFP steers compared to Control steers. Tumor necrosis factor-α is typically the first cytokine to be observed in circulation in response to an inflammatory insult, and it also stimulates the release of other pro-inflammatory cytokines, such as IL-6 and IFN-γ. Therefore, it is not surprising that there was a tendency for a reduction in IL-6. Extreme elevation of these pro-inflammatory cytokines can result in endotoxemia and death (Muchamuel et al., 1997); therefore, reduction in cytokine concentrations promotes survival. However, overall concentrations of TNF-α were relatively low compared to other studies measuring this cytokine in response to an LPS challenge, which may partially be explained by the dose of LPS administered in the current study (0.25 µg/kg) compared to others (1, 2, and 2.5 µg/kg; Waldron et al., 2003; Carroll et al., 2009). The reduction in the pro-inflammatory cytokine response may be a result of greater platelet and leukocyte concentrations observed in SCFP-supplemented steers. As the immune system was better prepared for interaction with a pathogen, this may have led to a reduction in the pro-inflammatory response as indicated in the reduction in pro-inflammatory cytokines, as well as serum cortisol concentrations. Additionally, the reduction in cytokines may have been affected by cortisol concentrations such that the greater initial cortisol response dampened the inflammatory cytokine response.

Acute phase proteins are indicative of infection and inflammation and increase in response to LPS challenge (Alsemgeest et al., 1994; Carroll et al., 2009). Various responses were observed in the acute phase protein response to the LPS challenge. No treatment differences were observed in haptoglobin concentrations, which is supported by work in finishing steers and dairy cows (Zaworski et al., 2014; Shen et al., 2019). While there was no overall treatment effect, SAA concentrations peaked later in SCFP-supplemented steers compared to Control. Zaworski et al. (2014) reported SAA concentrations in dairy cows supplemented with an SCFP were greater immediately after calving compared to control cows. Others have reported that SAA appears faster in serum compared to haptoglobin, suggesting that it is more acute in nature (Jacobsen et al., 2004). Perhaps a larger increase in haptoglobin would have been observed if additional samples were collected after the 24-h sample as haptoglobin appears to peak later (Jacobsen et al., 2004; Carroll et al., 2009). Fibrinogen, responsible for fibrin deposition during inflammatory events, was greater in SCFP steers throughout the study. The greater fibrinogen corresponds with greater platelet concentrations and a tendency for greater white blood cells, which express fibrinogen-binding sites (Smiley et al., 2001). Thus, the greater fibrinogen concentrations observed supports a potential priming of the immune system by SCFP.

## CONCLUSIONS

Calves supplemented with SCFP for 21 d prior to an intensive LPS challenge had greater rectal temperatures, platelets, glucose, and fibrinogen yet had reduced sickness behavior and reduced concentrations of the pro-inflammatory cytokines (TNF-α). Additionally, there was a delayed SAA response and a faster cortisol peak and recovery in SCFP-supplemented steers. This suggests that supplementing calves with SCFP may have primed the innate immune response prior to the challenge, particularly the immune cell populations and rectal temperature, which resulted in an attenuated sickness behavior and pro-inflammatory cytokine response to LPS. The combination of the innate immune system priming and the reduced inflammatory response may suggest that cattle may be better prepared for exposure to a pathogen.

## Supplementary Material

txaa156_suppl_Supplementary_Figures
