# Supplementary material for: Some aspects of the acute phase immune response to a lipopolysaccharide (LPS) challenge are mitigated by supplementation with a Saccharomyces cerevisiae fermentation product in weaned beef calves
Source: Transl Anim Sci. 2020 Aug 24;4(3):txaa156. doi: 10.1093/tas/txaa156 (PMC7575133; doi:10.1093/tas/txaa156)
Supplement: txaa156_suppl_Supplementary_Figures [file txaa156_suppl_supplementary_figures.docx]

**Supplementary Figures**

**Supplementary Figure S1**. Influence of supplementing steers with a *Saccharomyces cerevisiae* fermentation product (SCFP; 12 g·hd^-1^·d^-1^; *n* = 16) or not (Control; *n* = 16) for 21 d on the A) red blood cell; B) hemoglobin; C) hematocrit; and D) platelet response to lipopolysaccharide (LPS; 0.25 µg/kg BW) challenge. Complete blood counts were measured on whole blood samples collected every 2 h from -2 to 8 h and again at 24 h relative to LPS challenge at 0 h. There was an effect of time (*P* < 0.01) but no treatment or treatment × time interaction for red blood cells (A). There was a tendency (*P* = 0.10) for a treatment × time interaction for hemoglobin (B). Hematocrit was also affected by time (*P* < 0.01), but there was no treatment or treatment × time interaction. There was a tendency (*P* = 0.05) for platelet concentrations to be greater in SCFP-supplemented steers compared to control steers (D). Data presented as LSM ± SEM.

**Supplementary Figure S2**. Influence of supplementing steers with a *Saccharomyces cerevisiae* fermentation product (SCFP; 12 g·hd^-1^·d^-1^; *n* = 16) or not (Control; *n* = 16) for 21 d on the A) neutrophil; B) lymphocyte; C) neutrophil:lymphocyte ratio; D) monocyte; and E) eosinophil response to lipopolysaccharide (LPS; 0.25 µg/kg BW) challenge. Complete blood counts were measured on whole blood samples collected every 2 h from -2 to 8 h and again at 24 h relative to LPS challenge at 0 h. There was no effect of treatment or a treatment × time interaction for neutrophils, lymphocytes, neutrophil:lymphocyte ratio, or monocytes (A-D; *P* ≥ 0.16). There was a tendency (*P* = 0.09) for a treatment effect for eosinophils (E), where SCFP-supplemented steers tended to have greater concentrations than Control steers. Data presented as LSM ± SEM.

**Supplementary Figure S1**

**Supplementary Figure S2**
